# P-1694. Estimating the Use of Newer Beta-lactam Gram-negative Targeted Antibiotics Across Greater than 700 Hospitals in the United States

**DOI:** 10.1093/ofid/ofae631.1860

**Published:** 2025-01-29

**Authors:** Anthony Harris, Katherine E Goodman, Lisa Pineles, Morgan Walker, Jacqueline T Bork, Emily L Heil, Kimberly C Claeys, Justin Brooks, Sameer S Kadri, Brad Maron, Jonathan Baghdadi

**Affiliations:** University of Maryland School of Medicine, Baltimore, Maryland; University of Maryland School of Medicine, Baltimore, Maryland; University of Maryland School of Medicine, Baltimore, Maryland; Critical Care Medicine Department, Clinical Center, National Institutes of Health, Critical Care Medicine Branch, National Heart Lung and Blood Institute, Bethesda, MD; University of Maryland School of Medicine, Baltimore, Maryland; University of Maryland School of Pharmacy, Baltimore, MD; University of Maryland Baltimore, Baltimore, Maryland; University of Maryland, Baltimore County, Bethesda, Maryland; National Institutes of Health Clinical Center, Bethesda, Maryland; University of Maryland Institute for Health Computing, Bethesda, Maryland; University of Maryland School of Medicine, Baltimore, Maryland

## Abstract

**Background:**

Newer broad-spectrum beta-lactam antibiotics (cefiderocol, ceftazidime-avibactam, ceftolozane-tazobactam, imipenem-relebactam, meropenem-vaborbactam) have been introduced. Ceftazidime-avibactam and ceftolozane-tazobactam predominated newer antibiotic use through 2021; however, less is known about current use patterns and for what clinical indications, in inpatient settings. The aim of this study was to describe the use of these newer antibiotics across a large national cohort.

**Figure:**
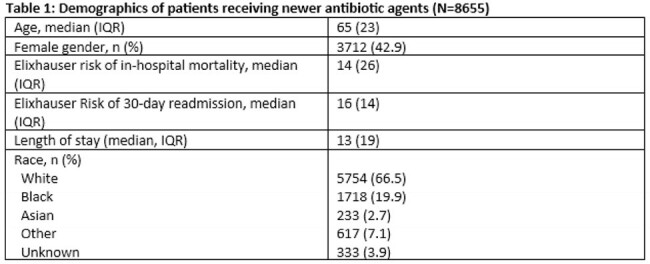

**Methods:**

We performed a retrospective cohort study of adults discharged from June 2022 through May 2023 among hospitals in the Premier Healthcare Database. Antibiotic utilization was ascertained from daily charge data, and clinical indication(s) were inferred from ICD-10 diagnosis codes. A stratified analysis was performed among patients receiving >3 days of antibiotic therapy to understand when these antibiotics are being used as definitive therapy.

**Figure:**
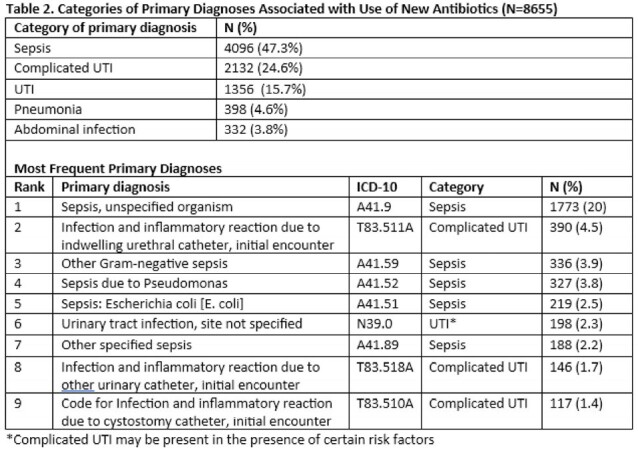

**Results:**

Across 832 hospitals, 3,890,557 admissions (61.9% of all admissions) included receipt of antibiotics. In 8,655 admissions (0.2% of antibiotic-prescribing admissions), newer antibiotics were prescribed (see Table 1 for patient demographic distribution). Ceftolozane-tazobactam was prescribed in 4157 (48%), 3660 (42.3%) ceftazidime-avibactam, 1060 (12.2%) cefiderocol, 456 (5.3%) meropenem-vaborbactam, and 99 (1.1%) imipenem-relebactam. Most patients who received newer antibiotics (69%, n=5,998) received them for greater than 3 days. Of these, ceftolozane-tazobactam was prescribed in 2607 admissions (43.5%), 2505 (41.7%) ceftazidime-avibactam, 832 (13.9%) cefiderocol, 332 (5.5%)meropenem-vaborbactam, and 68 (1.1%) imipenem-relebactam for greater than 3 days. Sepsis was the most common clinical indication for receipt of newer agents, followed by urinary tract infection (Table 2).

**Conclusion:**

Ceftazidime-avibactam and ceftolozane-tazobactam remain the most frequently prescribed new antibiotics, with uptake of subsequently approved agents trailing. Most of the prescribing of these antibiotics are for sepsis.

**Disclosures:**

**Anthony Harris, MD, MPH**, Innoviva: Advisor/Consultant|UpToDate: Infection Control Editor **Kimberly C. Claeys, PharmD, PhD**, bioMérieux: Advisor/Consultant|bioMérieux: Honoraria

